# Government spending, recession, and suicide: evidence from Japan

**DOI:** 10.1186/s12889-020-8264-1

**Published:** 2020-02-21

**Authors:** Tetsuya Matsubayashi, Kozue Sekijima, Michiko Ueda

**Affiliations:** 1 Osaka School of International Public Policy, Osaka University, 1-31 Machikaneyama, Toyonaka, Osaka, 560-0043 Japan; 2 Nippon Institute for Research Advancement, Yebisu Garden Place Tower, 34th Floor 4-20-3 Ebisu Shibuya-ku, Tokyo, Japan; 3 Faculty of Political Science and Economics, Waseda University, 1-6-1 Nishi-waseda Shinjuku, Tokyo, 169-8050 Japan

**Keywords:** Suicide, Government spending, Recession, Austerity, Japan

## Abstract

**Backgrounds:**

Austerity has been shown to have an adverse influence on people’s mental health and suicide rates. Most existing studies have focused on the governments’ reactions to a single event, for example, the Great Recession of 2008.

**Methods:**

This study focused on significant changes in fiscal policy between 2001 and 2014 in Japan. The size of expenditures by national and local governments decreased dramatically between 2001 and 2006 under the neoliberal reform and then increased after the global economic crisis and the Great East Japan Earthquake. Using the data from 47 prefectures between 2001 and 2014, we tested whether more spending by the local governments was associated with a lower suicide rate in their jurisdiction. We also investigated whether this relationship was particularly salient during a more severe recession.

**Results:**

Our analysis revealed that an increase of 1% in the per capita local government expenditures was associated with a decrease of 0.2% in the suicide rates among males and females aged between 40 and 64 and that this correlation was strengthened as the unemployment rate increased, particularly among males.

**Conclusions:**

Government’s reaction to economic crises can either exacerbate or mitigate the negative impact of the economic recession on people’s mental health and suicide rates.

## Background

The relationship between an economic downturn and people’s health has been extensively studied in a variety of disciplines. This topic has drawn increasing attention from scholars after the global economic crisis in 2008 [1–3]. Evidence on whether economic downturn improves or worsens people’s health is mixed, depending on the measures of health, demographic groups, levels of economic development, and the degree of downturns [1–4]. The results of previous studies are more consistent when we focus on mental health and suicidal risks as a measure of health: mental health worsens, and suicidal risks increase during the recession [5].

The adverse influence of the economic downturn on mental health and suicidal risks could be exacerbated or mitigated by government actions. This possibility became particularly evident in the aftermath of the 2008 Great Recession when many nations adopted fiscal austerity as a political reaction to the massive economic crisis [6–8]. Austerity in the period of the economic downturn can worsen people’s mental health in two major ways: by increasing economic insecurity among vulnerable individuals and by reducing healthcare services [9, 10]. Indeed, suicide rates increased after the Great Recession in countries where the austerity measures had been taken, including Greece, Ireland, Portugal, and Spain [11–17].

At the same time, the government can mitigate the effect of adverse economic shock by taking proper actions. For example, the amount of New Deal relief spending allocated to the US cities after the Great Depression between 1929 and 1940 was negatively correlated with the suicide rates of the area [18]. Similarly, the negative effect of the recessions on suicide rates was shown to be weakened in countries with relatively larger social welfare spending [19–21], though others reported no such relationship [22].

This study offers additional evidence on the role of the government’s actions to prevent suicide in the period of recession using longitudinal data from Japan. The study period is from 2001 to 2014, during which Japan experienced both the reduction and expansion of government expenditures under different administrations and political climates. More precisely, government expenditures significantly decreased under the neoliberal reform between 2001 and 2006 and then increased after the global economic crisis in 2008 and the Great East Japan Earthquake in 2011. In response to a severe recession that Japan experienced after the Asian financial crisis in the late 1990s, Junichiro Koizumi, who became the prime minister of Japan in 2001, adopted several major neoliberal reforms that downplayed the economic role of the government. Koizumi’s administration downsized the amount of government expenditures by up to 10% between 2001 and 2006, as compared to 2000. Such austerity measures, however, did not continue after Koizumi stepped down in 2006. The several administrations after Koizumi increased the amount of government expenditures mainly to stimulate economic activities after the Great Recession in 2008. In particular, Abe’s second administration (2012-) initiated various aggressive economic measures to recover from the long recession, including interventions that increased government spending on public infrastructure. In addition, the amount of public spending also expanded after the Great East Japan Earthquake in 2011 to mitigate its impact and to accelerate recovery from the disaster.

Thus, Japan has experienced both austerity and expansion as government policies over the last two decades. These policy changes at the national level also fundamentally affected the financial situation of subnational governments that relied heavily on fiscal transfers from the national government as a source of revenue. Prefectures and municipalities in Japan are administrative units that have independent sources of revenues, but about 30% of their total expenditures rely on a transfer from the national government. Thus, the amount of spending by local government crucially depends on the economic policies of the national government.

**Fig. 1:**
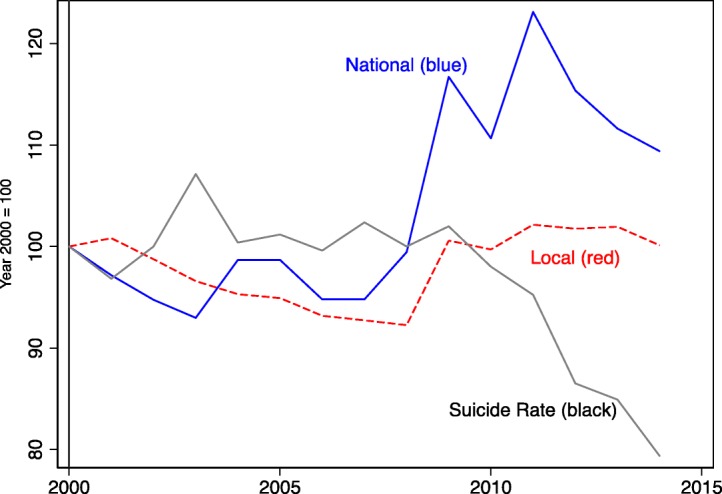
The change in the expenditures of the national and local governments and the suicide rate per 100,000 in Japan between 2001 and 2014, as the year of 2000 as a baseline (=100). Note: Data on the amount of expenditures were adjusted for inflation

In particular, when Koizumi’s administration lowered the number of transfers from the central government to subnational governments as part of his neoliberal reform, the volume of spending by subnational government declined, as shown in Fig. 1. The solid line in Fig. 1 depicts the change in the total amount of expenditure by the national government between 2001 and 2014 using the amount in 2000 as a baseline [23]. The dashed line in Fig. 1 depicts the total amount of spending by local governments [24]. The figure indicates that local government expenditures were highly correlated with national government expenditures and that local government expenditures decreased until 2007 and then increased. Notably, Fig. 1 also shows that the overall crude suicide rate shown as a solid gray line declined rapidly just after the amount of national and local government spending increased.

This study used these changes in the amount of local government spending associated with the policy changes at the national level in Japan to understand how the level of government expenditures affects suicide rates. Using data from 47 prefectures between 2001 and 2014, we tested two hypotheses: (1) Higher spending by the local governments was correlated with the lower suicide rates in their jurisdictions, and (2) the negative relationship between local government spending and the suicide rates was particularly strong during a more severe recession.

Our study improves upon previous studies on the role of government expenditures on suicide by focusing on different time horizons and types and levels of policymaking [25]. First, the evidence presented here does not concern only the government reaction to a single event, such as the studies focusing on the Great Recession of 2008. We examined the impact of various government actions over a period of 14 years. Second, this is the first study on the association between government spending and suicide in a non-European country, whereas the existing literature focused primarily on European countries and the United States. Third, our study is not a cross-country analysis, which often faces a challenge to isolate the effect of government spending from other national-level policy changes. Because our study used subnational variations over time, we were able to use unit-specific and year-specific fixed effects and thus control for unit-specific time-invariant and country-specific characteristics.

## Methods

We created panel data for 47 prefectures between 2001 and 2014. The number of observations was 658. We limited our data coverage until 2014 because of data availability. Using the panel data, we tested the first hypothesis that the larger amount of spending by subnational governments was correlated with the lower suicide rates in their jurisdictions by estimating the following model:

1 $$ {\left[S\right]}_{jt}=\alpha {\left[E\right]}_{jt}+\beta {\left[U\right]}_{jt}+\lambda {\mathbf{w}}_{jt}+{\mu}_jT+{\varphi}_t+{\rho}_j+{\varepsilon}_{jt} $$

where the outcome variable [S]_*jt*_ is a natural log of the suicide rate per 100,000 individuals in the year *t* in prefecture *j*. Suicide includes all deaths classified as X60-X84 under ICD-10. Considering the possibility that the effects of government expenditures varied by age and sex, we generated the suicide rates for six subpopulation groups: (1) males aged 20–39, (2) males aged 40–64, (3) males aged 65 and over, (4) females aged 20–39, (5) females aged 40–64, and (6) females aged 65 and over. The suicide data were calculated by using data from the Vital Statistics [26].

Our primary explanatory variable is [*E*]_*j*t_, which denotes the per capita government expenditures in prefecture *j* in the year *t*. We used the sum of expenditures of the prefectural government in *j* and all municipal governments in *j* from the Annual Report of Local Public Finance [24]. Japan is divided into 47 prefectures, and the number of municipalities in each prefecture ranges from 15 to 179. Both prefectural and municipal governments can use their expenditures on social welfare, public health, employment-related issues, public works, education, and disaster relief. We used the total amount of spending, rather than spending specifically for social welfare and public health because other types of local government spending can affect people’s well-being. For example, spending on infrastructure would produce job opportunities for the unemployed and may improve their economic and mental well-being. We transformed the per capita amount, adjusted for inflation, into a natural log for estimation.

[*U*]_jt_ in eq. (1) refers to the percentage of unemployed people in prefecture *j* in year *t*. Building on recent research on the same topic [21, 22, 27, 28], we used the unemployment rate as a measure of recession. We used the unemployment rate for the total population, though this data is limited in that it is not age-specific. The data were obtained from Statistics Japan [29].

Further, **w**_*jt*_ refers to the socioeconomic characteristics of each prefecture in each year, all of which were likely to affect both the suicide rate and the government’s expenditures. Specifically, included in **w**_*jt*_ are income per capita, fiscal strength index, population size, and percentages of the dependent population aged under 14 and 65 and over, in each prefecture and year. Income per capita, obtained from the System of National Accounts, was defined as the total amount of income in prefecture *j* in the year *t* divided by the population size [30]. The financial strength index measures the fiscal conditions of each prefecture each year. The index exceeds 1 if the amount of revenue coming from the prefecture’s financial sources exceeds the amount of fiscal demand and falls below 1 otherwise. This index is used to determine the amount of money transferred from the national to the local government. Because there were considerable year-to-year fluctuations, the values were averaged over the past 3 years. The data were obtained from the Annual Report of Local Public Finance [24]. The population size and the percentages of the dependent population were obtained from the Annual Resident Registers [31]. We used natural logs of the total population and income per capita in our regression analysis.

Finally, φ_*t*_ in eq. (1) represents the year fixed effects, while ρ_*i*_ represents the prefecture fixed effects unique to each prefecture. The year fixed effects allowed us to control for the effects of annual socioeconomic and political changes at the national level, such as the effects of macroeconomic policies and business cycle that might affect the entire country. It also controls for the effects of natural disasters such as the Great East Japan Earthquake in 2011. The prefecture fixed effects allowed us to control for the effects of time-invariant characteristics of the prefectures, such as the effects of culture related to suicide, and climate and geographic conditions. The inclusion of the prefecture fixed effects in eq. (1) means that the model used variations in the level of government expenditures over time within each local government. We also added the prefecture-specific linear time trends, μ_j_T, to the model to control for the effects of linear trends in the suicide rates unique to each prefecture.

To test the second hypothesis that the negative relationship between local government spending and the suicide rates was particularly robust during a more severe recession, we included the interaction term between [*E*]_*j*t_ and [*U*]_*j*t_ in the model as:

2 $$ {\left[S\right]}_{jt}=\alpha {\left[E\right]}_{jt}+\beta {\left[U\right]}_{jt}+\gamma {\left[E\right]}_{jt}\ast {\left[U\right]}_{jt}+\lambda {\mathbf{w}}_{jt}+{\mu}_jT+{\varphi}_t+{\rho}_j+{\varepsilon}_{jt} $$

Because the marginal effect of local government expenditures was hypothesized to change as the unemployment rate increased, we calculated and plotted the marginal effect of [*E*]_jt_ and its confidence interval at the different values of the unemployment rate.

## Results

The summary statistics of all variables used in our estimation are presented in Table 1. The suicide rates were higher among males than females. Males aged 40–64 showed the highest rate among all subgroups.

**Table 1:** Summary Statistics

	Mean	SD	Min	Max
Log of suicide rate: male 20–39	3.419	0.205	2.674	4.056
Log of suicide rate: male 40–64	3.926	0.257	3.287	4.684
Log of suicide rate: male 65-	3.811	0.214	3.132	4.453
Log of suicide rate: female 20–39	2.399	0.287	1.105	3.070
Log of suicide rate: female 40–64	2.638	0.213	1.733	3.183
Log of suicide rate: female 65-	3.000	0.296	1.749	3.926
Log of government expenditures per capita	13.658	0.212	13.102	14.393
Unemployment rate	4.257	1.029	2.100	8.400
Log of income per capita	14.848	0.153	14.506	15.541
Fiscal strength index	0.457	0.196	0.197	1.406
Log of population size	14.495	0.742	13.283	16.396
Percent under 14	13.848	1.111	10.786	19.879
Percent over 65	22.745	3.357	13.150	31.141
N of observations	658			

Note: Data covered the period from 2001 to 2014 in 47 prefectures of Japan

Table 2 reports the estimation results. Columns (1) to (3) report the results using the male suicide rates by three age groups, while columns (4) to (6) report the results using the female suicide rates. The prefecture-specific and year-specific fixed effects and the prefecture-specific linear time trend were always included in the models, but the estimates are not reported in the table. In order to address the potential heterogeneity and autocorrelation in the error terms within each prefecture, standard errors were clustered by prefectures.

**Table 2:** Estimated influences of government expenditures on suicide rates by sex and age

	(1) Male 20–39	(2) Male 40–64	(3) Male 65-	(4) Female 20–39	(5) Female 40–64	(6) Female 65-
Log of government expenditures per capita	−0.017	− 0.246	0.069	0.188	−0.207	− 0.043
	(−0.183, 0.150)	(− 0.428, − 0.063)	(− 0.080, 0.219)	(− 0.292, 0.669)	(− 0.383, − 0.032)	(− 0.322, 0.237)
Unemployment rate	0.014	0.023	0.062	−0.057	− 0.007	− 0.008
	(− 0.039, 0.067)	(− 0.017, 0.063)	(0.020, 0.105)	(− 0.110, − 0.004)	(− 0.078, 0.064)	(− 0.080, 0.064)
Log of income per capita	−0.266	− 0.173	−0.109	− 0.183	− 0.236	0.604
	(−0.988, 0.457)	(− 0.673, 0.327)	(− 0.630, 0.413)	(− 1.245, 0.879)	(− 1.143, 0.671)	(− 0.068, 1.276)
Fiscal strength index	− 0.026	0.038	0.107	− 0.518	− 0.055	− 0.397
	(− 0.323, 0.271)	(− 0.298, 0.375)	(− 0.146, 0.359)	(−1.245, 0.208)	(− 0.494, 0.383)	(− 1.078, 0.283)
Log of population size	1.396	3.446	1.881	10.457	0.976	2.042
	(−3.959, 6.750)	(−0.267, 7.160)	(−2.597, 6.360)	(−0.311, 21.225)	(−5.407, 7.359)	(−2.602, 6.686)
Percent under 14	0.149	−0.045	−0.095	0.170	−0.005	− 0.233
	(−0.061, 0.360)	(− 0.185, 0.095)	(− 0.289, 0.100)	(−0.160, 0.500)	(− 0.244, 0.233)	(− 0.436, − 0.029)
Percent over 65	0.154	0.044	0.017	0.049	−0.023	0.046
	(−0.012, 0.319)	(−0.085, 0.174)	(− 0.106, 0.139)	(− 0.176, 0.275)	(−0.215, 0.170)	(− 0.096, 0.189)
Prefecture fixed effect	Yes	Yes	Yes	Yes	Yes	Yes
Year fixed effect	Yes	Yes	Yes	Yes	Yes	Yes
Prefecture-specific liner trend	Yes	Yes	Yes	Yes	Yes	Yes
N of observations	658	658	658	658	658	658
R squared	0.342	0.825	0.405	0.262	0.269	0.448

Note: Table entries are regression coefficients with 95% confidence intervals in parentheses. Standard errors are clustered by prefectures. The dependent variable is a log of the suicide rate per 100,000 by sex and age. Data covered the period from 2001 to 2014 in 47 prefectures of Japan

Table 2 shows that the log of government expenditures per capita was negatively correlated with the log of suicide rates by males and females aged 40–64. More specifically, as the per capita expenditures decreased by 1%, the suicide rate among males aged 40–64 increased by 0.25% (95% CI: − 0.43, − 0.06) and the suicide rate among females aged 40–64 increased by 0.21% (95% CI: − 0.38, − 0.03). We found that the estimates were small or the confidence intervals were large in the other columns, suggesting that the local government’s expenditures had little relationship with the suicide rates by other sex and age subgroups.

**Fig. 2:**
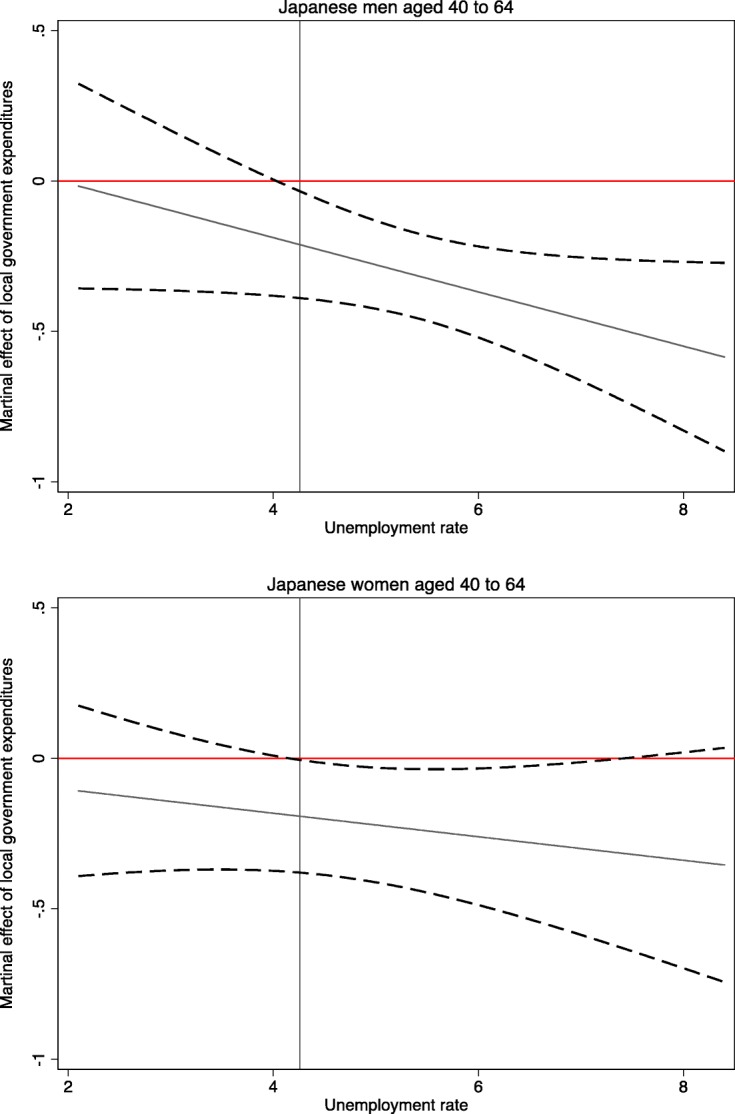
The marginal effect of government spending conditional on the unemployment rate in 47 prefectures of Japan. Note: These graphs are based on the estimation results in Table 3

Because the local government spending was shown to have a strong relationship only with the suicide rates of middle-aged males and females in Table 2, we focused on these two subgroups and estimated eq. (2). Using the estimated results reported in Table 3, we plotted the marginal effect of local government expenditures in a solid line and its confidence interval in a dashed line at the different values of the unemployment rate in Fig. 2. The vertical solid line in Fig. 2 denotes the average amount of government expenditures. The top panel shows that the negative relationship between local government expenditures and the log of suicide rate by males aged 40–64 was particularly relevant when the unemployment rate was modestly to extremely high. The confidence intervals overlap the horizontal line of zero when the unemployment was relatively low, suggesting that local government spending had a negligible relationship with the middle-aged male suicide rates when economic conditions are good. The bottom panel also indicates that the marginal effect of local government expenditures on the suicide rate of middle-aged females became larger as the unemployment rate was higher, but the change in the marginal effect over the scale of the unemployment rate was small.

**Table 3:** Estimated relationships between government expenditures and suicide rates conditional on the unemployment rate

	(1) Male 40–64	(2) Female 40–64
Log of government expenditures per capita	0.173	− 0.026
	(− 0.343, 0.688)	(− 0.458, 0.406)
Expenditures × unemployment	− 0.090	− 0.039
	(− 0.182, 0.002)	(− 0.126, 0.047)
Unemployment rate	1.256	0.528
	(−0.003, 2.515)	(−0.648, 1.703)
Log of income per capita	−0.153	− 0.228
	(−0.600, 0.295)	(−1.129, 0.674)
Fiscal strength index	−0.014	−0.078
	(−0.301, 0.272)	(−0.511, 0.354)
Log of population size	3.823	1.140
	(0.190, 7.457)	(−5.393, 7.672)
Percent under 14	−0.036	− 0.002
	(− 0.178, 0.105)	(− 0.243, 0.240)
Percent over 65	0.029	−0.029
	(−0.100, 0.158)	(−0.222, 0.163)
Prefecture fixed effect	Yes	Yes
Year fixed effect	Yes	Yes
Prefecture-specific liner trend	Yes	Yes
N of observations	658	658
R squared	0.827	0.269

Note: Table entries are regression coefficients with 95% confidence intervals in parentheses. Standard errors are clustered by prefectures. The dependent variable is a log of the suicide rate per 100,000 by men and women aged 40 to 64. Data covered the period from 2001 to 2014 in 47 prefectures of Japan

## Discussions

This study investigated the relationship between government expenditures and suicide rates by using the variations in the amount of local government spending associated with the national-level economic policy change. Our estimation models controlled for the effects of other relevant factors, such as the unemployment rate and per-capita income, as well as the prefecture- and year-specific factors and time trends. We found that the suicide rates of middle-aged males and females tended to increase when prefectural governments decreased their spending level. This negative association was stronger among males when the unemployment rate increased. These findings suggest that local suicide rates could be reduced when local governments increase their spending. The magnitude of the effect is not trivial. During our study period, the average male suicide rate for the middle-aged was 31.19, and a one-percent increase in government spending would translate to a reduction of the suicide rate per 100,000 by 0.078 cases, which translates to 0.36 cases of suicide in each prefecture and 17 suicides across the whole of Japan per year.

However, we also found that the amount of spending by subnational governments had little relationship with the suicide rates of the younger generation and those of the elderly population. The null-finding for the elderly group is not surprising, as they are less likely to be working and, hence, less likely to benefit from any increased economic activities associated with an expansion in government spending. However, they are also more likely to be beneficiaries of welfare-related spending, and thus the finding is somewhat counterintuitive at the same time. As for the younger generation, it is possible that their suicide rates are determined by an entirely different set of factors. According to the data compiled by the National Police Agency, among those whose motives and reasons behind their suicide are known, 3 and 14% of suicide deaths by those aged less than 20 and aged 20–29 are related to economic hardship, respectively, whereas 19 and 22% of deaths by those aged 30–39 and 40–49 are attributable to economic conditions, respectively [32]. Among the elderly population (ages 70 and higher), a majority of their suicides is due to health-related reasons, and only 6% of their suicides are due to economic factors.

We conducted robustness checks to ensure that our results are not sensitive to the specific model and data that were chosen. The above results were based on the total amount of government spending of both prefectural and municipal governments combined. We evaluated the same models with the total amount of government expenditures only by prefecture governments. The main results did not change. In order to check if the expenditures in the previous year matter for the well-being of the population, we also took a one-year lag of local government’s expenditures and added the lagged value as an additional regressor. We found that the amount of government spending in the previous year does not affect the suicide rate; the estimated results suggest that only the government expenditures in the current year affect the suicide rate for that year. Both results are available upon request.

The present study contributes to the existing literature by providing further evidence on the importance of government actions on suicide rates. Depending on how the government reacts to economic shocks, it can either exacerbate or mitigate the negative impact of the economic recession on the population. The findings of this study are mostly consistent with those of other studies that showed that austerity measures during an economic crisis could have a detrimental effect on people’s mental health and health in general.

The major strength of this study lies in the fact that we examined the effect of different government reactions to multiple economic crises over an extended period within a single country. Most previous studies have examined the impact on people’s health of government reactions to a single major financial crisis, such as the 2008 Great Recession, by using a cross-national comparison. Our analysis of the sub-national data in Japan allowed us to isolate the effect of government spending from other major national-level policy changes, which thus provided more robust evidence for the relationship between austerity and suicide. Moreover, to our knowledge, this is the first study on the association between government spending and suicide in a non-European country.

This study has several limitations. First, our study cannot provide answers as to why increased government spending can decrease the number of suicides as our study was ecological in nature. Second, our analysis did not explicitly consider the potential effect of various suicide prevention measures on suicide rates. The Japanese government introduced its first national suicide prevention program in the mid-2000s, and the suicide rates have decreased since around 2012. The prefecture fixed effects and the prefecture linear trends that we included in the model should capture most of the effects of suicide prevention measures. However, in order to check this possibility more explicitly, we also added the per capita amount of transfers from the national government earmarked for suicide prevention activities to model (1) to control for the effect of local suicide prevention activities. We found that our main results did not change by this modification and also that the amount spent on suicide prevention activities does not seem to affect suicide rates in a meaningful way. The results of this supplementary analysis are reported in Table 4.

**Table 4:** Estimated influences of total government expenditures and expenditures for suicide prevention programs on suicide rates by sex and age between 2001 and 2014 in 47 prefectures of Japan

	(1) Male 20–39	(2) Male 40–64	(3) Male 65-	(4) Female 20–39	(5) Female 40–64	(6) Female 65-
Log of government expenditures per capita	− 0.018	−0.242	0.075	0.186	−0.201	− 0.047
	(−0.185–0.149)	(−0.426 - -0.059)	(− 0.076–0.227)	(−0.295–0.667)	(−0.384 - -0.019)	(− 0.318–0.224)
Log of government expenditures per capita	−0.001	0.003	0.006	−0.002	0.006	−0.004
for suicide prevention programs	(−0.006–0.004)	(−0.001–0.008)	(0.001–0.010)	(−0.010–0.005)	(0.001–0.011)	(−0.012–0.004)
Unemployment rate	0.014	0.023	0.061	−0.057	−0.008	− 0.008
	(−0.039–0.068)	(−0.017–0.062)	(0.019–0.103)	(−0.110 - -0.004)	(− 0.079–0.064)	(−0.081–0.065)
Log of income per capita	−0.268	− 0.168	−0.100	− 0.186	−0.228	0.598
	(−0.991–0.456)	(−0.665–0.328)	(−0.618–0.418)	(− 1.251–0.878)	(− 1.140–0.685)	(− 0.069–1.266)
Fiscal strength index	−0.033	0.053	0.134	−0.528	−0.028	− 0.415
	(−0.338–0.272)	(−0.286–0.392)	(−0.119–0.387)	(−1.264–0.207)	(−0.473–0.417)	(−1.104–0.273)
Log of population size	1.251	3.776	2.475	10.237	1.562	1.655
	(−4.237–6.740)	(−0.011–7.563)	(−1.995–6.945)	(−0.742–21.216)	(−5.239–8.362)	(−2.831–6.140)
Percent under 14	0.149	−0.045	− 0.095	0.170	− 0.005	−0.233
	(−0.061–0.360)	(−0.185–0.094)	(−0.289–0.099)	(−0.161–0.501)	(−0.245–0.234)	(−0.437 - -0.028)
Percent over 65	0.153	0.046	0.019	0.048	−0.021	0.045
	(−0.012–0.318)	(−0.084–0.175)	(−0.103–0.141)	(−0.177–0.274)	(−0.214–0.172)	(−0.098–0.187)
Prefecture fixed effect	Yes	Yes	Yes	Yes	Yes	Yes
Year fixed effect	Yes	Yes	Yes	Yes	Yes	Yes
Prefecture-specific liner trend	Yes	Yes	Yes	Yes	Yes	Yes
N of observations	658	658	658	658	658	658
R squared	0.342	0.825	0.407	0.262	0.270	0.448

Note: Table entries are regression coefficients with 95% confidence intervals in parentheses. Standard errors are clustered by prefectures. The dependent variable is a log of the suicide rate per 100,000 by sex and age. Data covered the period from 2001 to 2014 in 47 prefectures of Japan

## Conclusion

In conclusion, our findings suggest that government actions can significantly affect the health of the general public, even if the actions are not directly related to spending for public health and social security. Decreasing the level of *overall* government spending can be detrimental to people’s health.

## Data Availability

The datasets used and/or analyzed during the current study are available from the corresponding author on reasonable request.
